# Computer Aided Detection of Breast Density and Mass, and Visualization of Other Breast Anatomical Regions on Mammograms Using Graph Cuts

**DOI:** 10.1155/2013/205384

**Published:** 2013-09-10

**Authors:** Nafiza Saidin, Harsa Amylia Mat Sakim, Umi Kalthum Ngah, Ibrahim Lutfi Shuaib

**Affiliations:** ^1^Imaging & Computational Intelligence Group (ICI), School of Electrical and Electronic Engineering, Universiti Sains Malaysia, Engineering Campus, 14300 Nibong Tebal, Pulau Pinang, Malaysia; ^2^Advanced Medical and Dental Institute, Universiti Sains Malaysia, No 1-8 (Lot 8), Persiaran Seksyen 4/1, Bandar Putra Bertam, 13200 Kepala Batas, Pulau Pinang, Malaysia

## Abstract

Breast cancer mostly arises from the glandular (dense) region of the breast. Consequently, breast density has been found to be a strong indicator for breast cancer risk. Therefore, there is a need to develop a system which can segment or classify dense breast areas. In a dense breast, the sensitivity of mammography for the early detection of breast cancer is reduced. It is difficult to detect a mass in a breast that is dense. Therefore, a computerized method to separate the existence of a mass from the glandular tissues becomes an important task. Moreover, if the segmentation results provide more precise demarcation enabling the visualization of the breast anatomical regions, it could also assist in the detection of architectural distortion or asymmetry. This study attempts to segment the dense areas of the breast and the existence of a mass and to visualize other breast regions (skin-air interface, uncompressed fat, compressed fat, and glandular) in a system. The graph cuts (GC) segmentation technique is proposed. Multiselection of seed labels has been chosen to provide the hard constraint for segmentation of the different parts. The results are promising. A strong correlation (*r* = 0.93) was observed between the segmented dense breast areas detected and radiological ground truth.

## 1. Introduction

Breast cancer is the most frequently diagnosed cancer (about 23% (1.38 million) of total cancer cases) and the leading cause of cancer deaths amongst females worldwide (14% (458,400) of total cancer deaths), in 2008 alone [1]. One of the reasons for the increase in incidences and mortality rate of breast cancer is due to the lack of awareness of the disease and poor response to calls for breast screening. Early detection through mammography has been shown to increase treatment options and save lives. Women with high breast density are more likely to be afflicted with breast cancer, that is, about four to five times than women with low breast density [2, 3]. Mammography is the only imaging technique that has the ability to detect breast cancer even before it becomes palpable. Mammograms are analyzed visually by radiologists. However, there are limitations since the sensitivity of mammography is reduced on dense breasts [4]. Hence, there is difficulty in the interpretation of such mammogram images. Because of the subjective nature of visual analysis, qualitative responses may vary from one radiologist to another. As a solution, a computerized system which can detect the dense breast areas and act as second opinion is essential. 

Segmentation of abnormal structures in the breast, consequently, depends on breast tissue density. According to Suckling et al. [5], automated segmentation of glandular tissue or parenchymal pattern can be provided as a primer for mammographic lesion detection. A mass is usually represented by a hyperdense structure. However, overlapped fibroglandular tissues also appear to have similar intensities with a mass [6]. According to Miller and Astley [7], identification of glandular tissue in a mammogram is necessary for assessing asymmetry between the left and right breasts. Matsubara et al. [8] have stated that the assessment of fibroglandular tissue which can be used to estimate degree of risks is obscured by normal breast tissues. It is difficult to differentiate between normal, dense tissue, and cancerous tissue when the tumor is surrounded by glandular tissues [9]. 

In clinical practice, Caulkin et al. [10] have realized that the majority of cancers are associated with glandular rather than the fatty tissues. The detection of breast cancer in mammograms is a very difficult task. This is due to the large variation of breast tissues appearance in mammograms. Tumor or mass is usually represented by dense tissues located on the fibroglandular region and can often be seen as light gray or bright regions in the mammograms. Hence, for mammograms which are dense and of glandular type, the detection of tumors becomes more difficult because of the similarity of intensities between the tumor and the dense normal breast tissues. The detection of tumor that is embedded in the normal dense tissue thus becomes more complex compared with the fatty breast tissues. 

Many researchers had focused on image processing, including segmentation techniques to identify masses and calcifications for the detection of early breast cancer. Most of the image processing techniques are implemented on the whole mammogram without taking into consideration that mammograms have different density patterns and the fact that anatomical regions are used by radiologists in the interpretation [6]. Segmentation of the glandular tissue can act as a primary step to detect suspicious mass and to reduce false positives. The focus of this research is not only for the segmentation of dense areas but also to consider the existence of mass or masses and other breast anatomical structures for visualization. Hence, by focusing on the glandular areas and highlighting the hyperdense regions of the glandular area, it can assist and contribute as a second opinion for experts in diagnosis. 

In this study, GC technique is explored to evaluate its efficiency to detect breast density and masses, and to visualize other breast anatomical regions on mammograms. GC technique enables objects in medical images to be reliably segmented by finding their precise boundaries. Existing research on GC technique research papers has shown positive results in the segmentation of medical images such as magnetic resonance imaging (MRI) and computed tomography (CT) images. Although the GC techniques have shown very promising outcome, work on using the technique applied on mammograms has yet to embark. Camilus et al. [11] used the GC algorithm to identify the pectoral muscle. Our previous work [12] was the first to have used GC algorithms on mammograms to segment breast regions into section of background, skin-air interface, uncompressed fat, compressed fat, and glandular regions. The niche of this study is the application of the GC technique for detecting breast density, mass, including the breast anatomical regions on mammograms. We have found that it is important to combine segmentation of the breast into anatomical regions with the segmentation of breast density for general breast cancer screening. Then, focusing on the dense components and specific segmentations of glandular tissue areas should be adapted for breast mass detection. Finally, breast density estimation for breast cancer risk assessment or for monitoring the changes in breast density as prevention or intervention procedure should also be incorporated. Therefore, the steps in the Computer Aided Detection System would be combined and be of use in this work. 

## 2. Background

Wolfe [13] was the first to have shown the relationship between mammographic breast density patterns and the risk of developing breast cancer. He classified breast density or described it as parenchymal patterns into four categories. Because of the relationship, studies based on breast density have been undertaken. Miller and Astley [7] investigated texture-based discrimination between fatty and dense breast types. Byng et al. [14] used measures based on fractal dimension. Zhou et al. classified breast density into one of four BIRADS categories according to the characteristic features of gray level histogram [15]. They found that the correlation between computer-estimated percent dense area and radiologist manual segmentation was 0.94 and 0.91 with root-mean-square (RMS) errors at 6.1% and 7.2%, respectively, for CC and MLO views. Matsubara et al. [8] divided breast mammogram images into three regions using variance histogram analysis and discriminant analysis. Then, they classified it into four categories, which are (1) fatty, (2) mammary gland diffuseness, (3) nonuniform high density, and (4) high density, by using the ratios of each of the three regions.

Bovis and Singh [16] estimated features from the construction of spatial gray level dependency matrices. Petroudi et al. [17] used textons to capture the mammographic appearance within the breast area. Several other researchers used intelligence systems for density classification such as probabilistic Latent Semantic Analysis (pLSA), *k*-nearest neighbors (kNN) classifier, a decision tree classifier, and a Bayesian classifier. Bosch et al. [18] proposed a new approach to model and classify breast parenchymal tissue using pLSA. Chatzistergos et al. [19] worked on the classification of breast tissue according to Breast Imaging Reporting and Data System (BIRADS) using pLSA. Oliver et al. [20] used the k-nearest neighbors (kNN) classifier, a decision tree classifier, and a Bayesian classifier, based on the combination of the first two classifiers in their research.

Despite all the studies, only a small group of researchers have been involved with the segmentation of dense breast areas with regards to the breast anatomical structure. More detailed divisions can be made through segmentation based on the anatomy. This also helps in the delineation, characterization, and visualization. For example, with the detection of the breast region, other related clues such as distortion in breast structure and the nipple position in the breast will also be detectable. The segmentation method proposed by Karssemeijer [21] allowed the subdivision of a mammogram into three distinct areas: breast tissue, pectoral muscle. and background. Petroudi and Brady [22] described an algorithm to segment mammographic images into regions corresponding to different densities. The segmentation algorithm used textons in a hidden markov random Field (HMRF). The results of the algorithm demonstrated close agreement to radiologist's segmentation and density interpretation. Adel et al. proposed segmentation of breast regions into pectoral muscle, fatty, and fibroglandular regions using Bayesian techniques with an adaptation of Markov random field for detecting regions of different tissues on mammograms [23]. Aylward et al. segmented the breast into five regions using a combination of geometric (gradient magnitude ridge traversal) and statistical (Gaussian mixture modeling) methods [24]. The five regions that they segmented are the background, uncompressed fat, fat, dense tissue, and muscle. El-Zaart segmented the mammogram image into three regions, which are fibroglandular disc, breast region, and background [25]. In many segmented images, the outline of the breast region is positioned more inwardly than the actual boundary, perhaps because the skin line was hardly visible. The previous segmentation research by Oliver et al. resulted in a minor loss of skin-air regions in the breast area [20]. There is a need to build a system that attempts to avoid this situation by preserving the skin line position and, if possible, nipple location. This is important because it assists the practitioner in the detection of architectural distortion. Radiologist gives specific attention to the nipple in physical examination of the breast. Moreover, according to Karssemeijer [21], it is important to preserve the skin line position for feature selection. In our previous work, GC technique is found to be able to segment the breast regions into the background, skin-air interface, fatty, glandular, and pectoral muscle [26].

Most of the studies are focused on the classification methods for breast density. Others highlighted the segmentation methods for fibroglandular tissue, while few researchers performed segmentation of the breast anatomical regions based on density. There have also been works on the segmentation of other specific parts of breast regions such as either detection of nipple position, skin-air interface, or pectoral muscles. Breast density research had been reviewed intensively in our previous paper [27]. Most of the previous research paper that focused on the segmentation of the glandular area do not usually emphasize on the ability of tumor detection. This study not only segments the dense breast areas but also detects tumor in that area. It would be interesting if, at the same time, the system can visualize the breast anatomical regions in order to assist radiologist in the interpretation. As a continuation of previous work [26], this study will highlight the capability of GC technique not only for breast density and other breast anatomical regions segmentation but also considering the detection of mass or masses. Pectoral muscle extraction is also carried out in order to perform breast density classification. Our research goal is to develop a system which can detect breast density and mass, and visualize other breast regions (skin-air interface, fatty, glandular, and pectoral muscle) in a system. The performance evaluation of the breast density segmentation results and a model to estimate BIRAD category according to breast density percentage in the classification stage will be presented in this paper.

Although there are other researchers who segment the breast region into fatty and dense regions [23, 25], the technique in this study has the capability of segmenting the image into its anatomical regions up to six regions. There are also studies which concentrate on the detection of breast boundary or localization of nipple [28]. However, they have not considered the detection of dense areas. On the other hand, previous works which concentrate on the detection of breast density have not considered the preservation of breast boundary or localization of nipple [20]. Our approach here would take into consideration the detection of breast density as well as to preserve as much as possible, the breast boundary or localization of nipple position. We present a method for segmenting the breast to areas of different densities which investigates the use of GC algorithm. The results of the segmentation are based on user defined seed labels depending on the density features to provide hard constraints for the GCs algorithm, as they combine tissue type and color information.

## 3. Methodology

**Figure 1:**
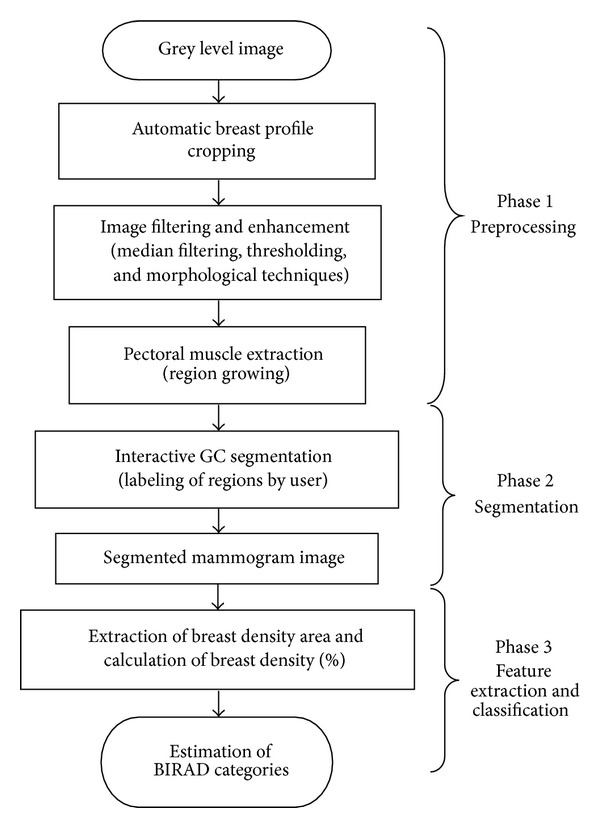
Block diagram representing each phase involved in this study.

The main objective of this study is to develop a Computer Aided Detection System, which is capable of segmenting breast density and mass, and also visualizating other breast anatomical regions. Several steps are involved to achieve this objective. All the phases involved have been shown in the block diagram of Figure 1. The initial steps in this research involved the preprocessing stage, including automated image cropping, artifacts removal, and image enhancement. In this study, GC techniques are proposed for the segmentation of dense areas and mass, and visualization of other breast anatomical regions. The method has been tested on the Mammographic Image Analysis Society Digital Mammogram Database (Mini-MIAS) database [29]. 

### 3.1. Preprocessing Step

Automated breast profile cropping is performed to locate the region of interest (ROI) containing the desired breast region. In order to save the usage of memory space and to speed up the processing task, the image has been down sampled by 4. The image cropping removes unnecessary areas such as the wide background areas and allows more focus on specific regions. The purpose of this procedure is to enable the process of suspicious area detection to be limited to the breast area without being influenced by the background. This ROI will be used for further segmentation processes. 

Some images in mini-MIAS database are affected by artifacts and noises. Artifacts in the mammogram images are of high intensity such as labels, opaque marker, and scanning artifacts. Noises such as speckled noises and scratches are most likely to be caused by the digitization process. The opening morphological and thresholding technique is employed at the preprocessing stage to remove the artifacts and noises, and to ease the segmentation process. The noise and artifacts in the background are detected and replaced by black pixels. For enhancement, the image is processed using median filtering and morphological techniques. A median filter is used because of its ability to remove artifacts caused by scratches.

### 3.2. Pectoral Muscle Extraction

The similarity in intensity and the overlap between the pectoral muscle and the glandular tissue can cause false positive detection of mass or dense area. Extraction of the pectoral muscle area can help to reduce the false positives. Removing the pectoral muscle is also essential in calculating the percentage of breast area. This is an improvement of our previous research [12, 26, 27, 30, 31], which only considered the detection of dense areas and visualization of other anatomical regions. In this research paper, calculation of breast density percentage will be carried out, involving removal of pectoral muscle region in the calculation of breast region area. The procedures involved in the detection of pectoral muscle are as foloows:

1. the result from the automatic breast profile cropping image will be used further in this stage. The left MLO mammogram image is flipped horizontally to position the pectoral muscle at the upper-left corner of the image. While the right MLO mammogram image can be processed directly without the need for flipping;
2. the initialization seed of pectoral muscle is automatically located at the upper-left corner of the initial location for region growing;
3. the identified pectoral muscle area using region growing is extracted and removed;
4. the breast region without pectoral muscle is used as input for segmentation using GC technique.

### 3.3. Multilabel Graph Cuts Segmentation

It is difficult to identify breast density due to the fuzzy boundary between fatty and glandular regions. Thus, it is very necessary to segment the different kinds of tissues or breast anatomical structures in the mammogram image for accurate diagnosis. Image segmentation using GC is used to partition an image according to the breast anatomical structure and to allocate the dense breast areas or tumor. The GC is applied with a multiselection of seed labels to provide the hard constraint, whereas the seeds labels of different breast regions are user defined selected. This method is essential in mammogram image processing in order to identify the dense breast areas as well as the abnormal locations. The precision of this segmentation technique has a great effect on image analysis. 

The segmentation result using GC is performed using the following equation:

(1) $$\begin{array}{c} E\left(f\right)=\lambda \cdot \overset{}{\underset{p\in P}{\sum }}D_{p}\left(L_{p}\right)+\mu \cdot \overset{}{\underset{\left\{p,q\right\}\in N}{\sum }}V_{\left\{p,q\right\}}\cdot \delta \left(L_{p}\neq q\right). \end{array}$$

*E*(*f*) is an energy function, and *L* = {*L*_*p*_ | *p* ∈ *P*} is a labeling of image *P*. The first term of this equation is called data the cost (also known as the regional properties term) [32], while the second term is called smooth cost (also called boundary properties term). *D*_*p*_(·) is a data penalty function, and it indicates individual label preferences of pixels based on observed intensities and prespecified likelihood function. *V*_*p*,*q*_ is interaction potential which encourages spatial coherence by penalizing discontinuities between neighboring pixels. There are two constants; *λ* and *μ* correspond to datacost and smoothcost, purposely to obtain the optimal segmentation. In this work, the value for data cost constant (*λ*) is set to 10 and smooth cost constant (*μ*) is set to 20. These values were chosen because the best segmentation results were obtained based on a trial and error basis.

In using graph cuts, the user only needs to select the number of segments and also put labels on the desired regions to perform the segmentation. To represent meaningful anatomical regions, three to six numbers of segments are appropriate. The initial labels that need to be assigned are labels for the background and skin-air interface, which will separate breast and nonbreast regions in the mammogram. Then, the most important label is for the dense and hyperdense regions which have a higher possibility of harbouring the suspicious region. Tumors or masses are usually represented by hyperdense structures embedded in the dense part of the glandular tissue. Hence, by focusing on the glandular area and highlighting the hyperdense region of the glandular area, the GC algorithm can automatically detect the abnormal area. As output, the mammogram image is presented as a number of segments, with each segment representing the different regions of the breast.

### 3.4. Performance Evaluation

Performance evaluation step is carried out to measure the capability of the proposed method. The most important part that we want to highlight in this study is the density area. Three performance metrics used in the performance evaluation in previous studies [28, 33] are completeness (CM), correctness (CR), and quality (*ρ*) [23]. The completeness is the percentage of the ground truth region which is explained by the segmented region. The correctness is the percentage of correctly extracted breast region type. A single metric which is quality, can be obtained by combining completeness and correctness [33]. The optimum value for both metrics is 1. These same three performance metrics evaluations are also used in this study, which are as follows:

(2) $$\begin{array}{c} \text{Completeness}\approx \frac{\text{TP}}{\text{TP}+\text{FN}},\\ \text{Correctness}\approx \frac{\text{TP}}{\text{TP}+\text{FP}},\\ \text{Quality}\approx \frac{\text{TP}}{\text{FN}+\text{FP}+\text{TP}}. \end{array}$$

The terms and formula involved in evaluating segmentation results are stated as folows:

1. true positive (TP) means the pixels correctly segmented as glandular tissue/mass that proved to be glandular/dense tissue in the ground truth;
2. false positive (FP) means the pixels correctly segmented as glandular tissue/mass that proved to be other tissues in the ground truth;
3. false negative (FN) means the pixels correctly segmented as other tissues that proved to be glandular tissue/mass in the ground truth;
4. true negative (TN) means the pixels correctly segmented as other tissues that proved to be other tissues in the ground truth.

**Figure 2:**
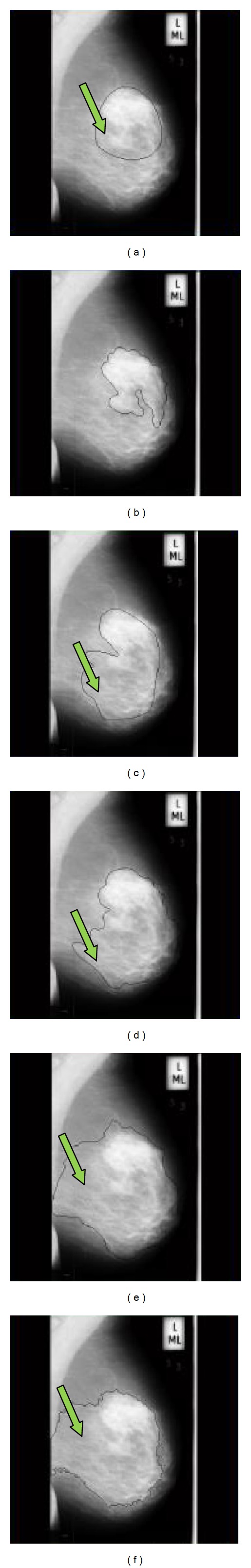
Ground truths from radiologists for mammogram image mdb111 [34].

When analyzing mammography images in the screening process, one of the tasks of the radiologist is to identify the portion of image that represents breast density area. This is very subjective. Different experts sometimes have differing opinions even when referring to the same image.  Figure 2 is the ground truth of breast density showing the variability in the interpretation of breast density or glandular tissue amongst radiologists [34]. As we can see from Figure 2, the breast density is represented by one location spread over a large area as their ground truths. The green arrows showed that fatty tissues were also included inside the ground truth of the breast density. 

Most of the research done in density detection does not provide comparison with expert delineation of density area. In fact, qualitative analysis was always needed as performance evaluation [35]. In this research, segmented images using GC are compared with manually sketched sections of dense breast areas using MIPAV program by an expert radiologist. The segmented density area by the radiologist becomes the gold standard of reference for measuring the validation of our method. This can provide more accurate measurements of breast density.

**Figure 3:**
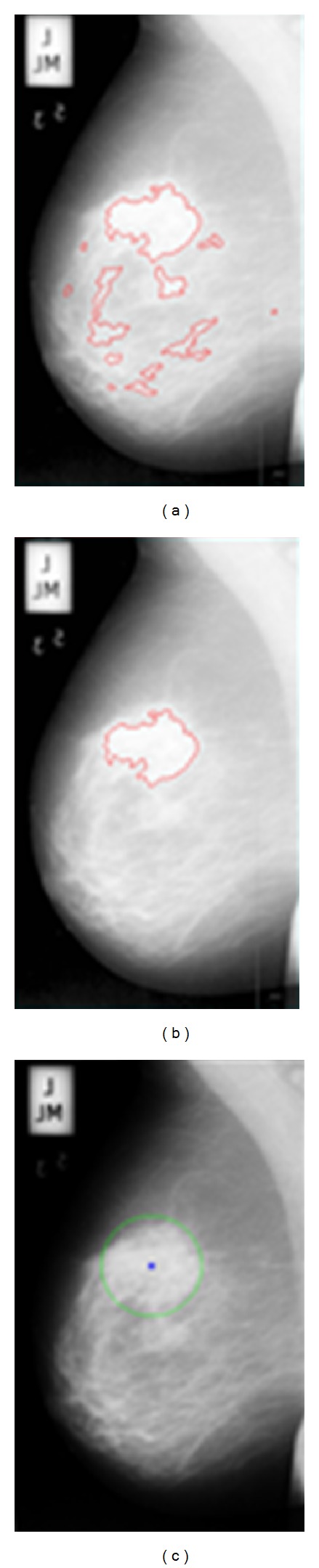
Ground truth from radiologist for (a) breast density and (b) mass area; (c) ground truth from mini-MIAS database for mass area.

Information on the location of abnormality and the radius of a circle enclosing the abnormality that can be used as reference for mass detection have been provided in the mini-MIAS database. In this study, a procedure has been created whereby the location of abnormality can be inserted together with the radius of a circle enclosing the abnormality and also automatically detects the location of abnormality. The centroid location of abnormality is represented by a blue asterisk, while a green circle represents the abnormality enclosed as shown in Figure 3(c). 

**Figure 4:**
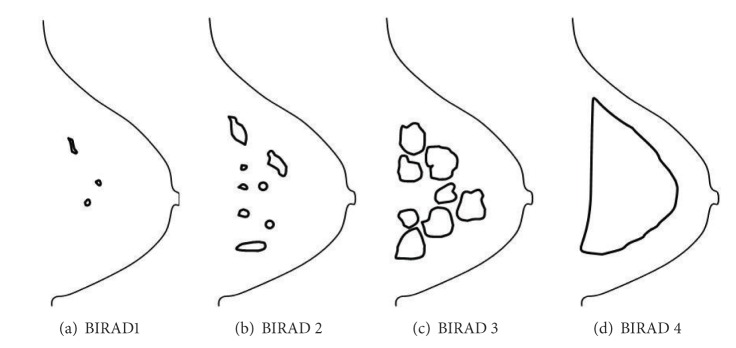
The illustrations of breast density ground truth by radiologist for each BIRAD category.

However, for quantitative analysis, the ground truth of the abnormality is also needed. This requires manual sketchings from the radiologist expert to highlight the edges of density and mass area. In this study, the process is confined to one single best expert opinion, that is, a senior radiologist who is also a consultant radiologist, having more than 20 years of experience. In his opinion, one sketching for a ground truth is appropriate to represent a homogeneous region (BIRAD 1 or BIRAD 4). However, a heterogeneous region (BIRAD 2 or BIRAD 3) is not appropriately represented by one sketching of the ground truth. This is because, by using a rough ground truth to represent the heterogeneous region, the fatty tissue would most probably be also included inside the regions such as the green arrow labeled ground truths as in Figure 2. According to our expert, since the heterogeneous region greatly differs from a homogeneous region, more than one sketching is required for ground truths of this nature. In this study, the ground truths were obtained to locate the dense breast regions and mass areas, which are indicated by red lines as in Figures 3(a) and 3(b). These can be used as basis of references for comparing the validity of our segmentation results.  Figure 4 gives the illustration of ground truths by our expert for different BIRAD categories. Thus, more appropriate segmentations may be obtained from this study as a basis of comparison using our computerized method. 

### 3.5. Classification of Breast Density

**Figure 5:**
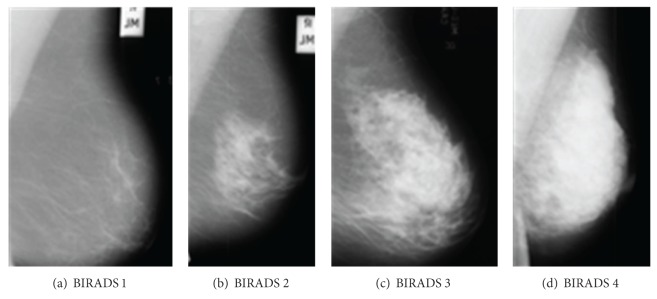
Mammograms images according to BIRADS categories.

Breast Imaging Reporting and Data System (BIRADS), which was developed by the American College of Radiology (ACR) is the recent standard in radiology for categorizing breast density [36]. BIRADs classify breast density into four major categories: (1) predominantly fat (<25% fibroglandular content); (2) fat with some fibroglandular tissues (fibroglandular content between 26% and 50%); (3) heterogeneously dense (fibroglandular content between 51% and 75%); and (4) extremely dense (fibroglandular content > 75%).  Figure 5 shows mammogram images with different BIRAD categories. Breast density percentage is calculated using the following formula [37]:

(3) $$\begin{array}{c} \text{Breast}\;\text{density}=\frac{\text{Glandular}\;\text{region}}{\text{Breast}\;\text{region}}\times 100\%.\;\;\; \end{array}$$

Breast density percentage was calculated by dividing the number of density pixels by the total number of pixels within the breast boundary. A different breast density category is obtained by grouping the breast density percentage calculated according to the BIRAD categories. A statistical analysis using Pearson correlation coefficient [38] will be used for comparison of the breast density area using GC segmentation method and the breast density area derived by the ground truth. Regression analysis will also be carried out to determine a model for prediction of BIRAD categories in this classification stage.

## 4. Results

**Figure 6:**
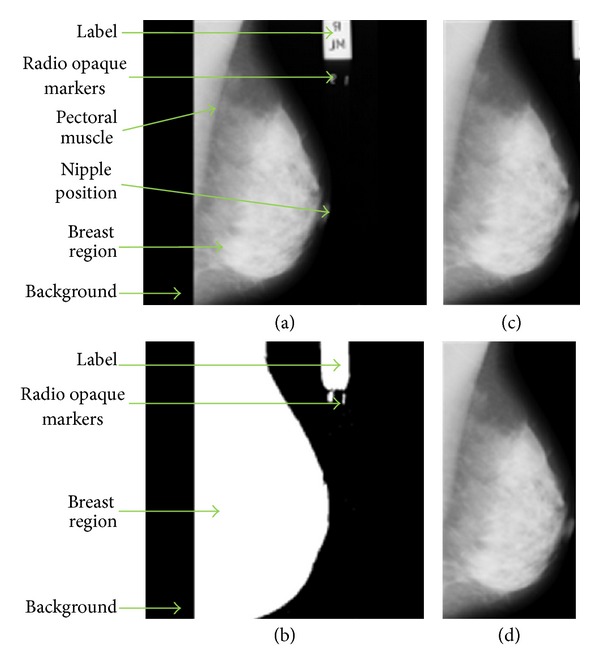
(a) Original mammogram image of mdb004; (b) binarized image of the breast region after thresholding; image after preprocessing using the median filter and automatic cropping (c) before and (d) after label and marker removal resulting in the segmented breast profile.

**Figure 7:**
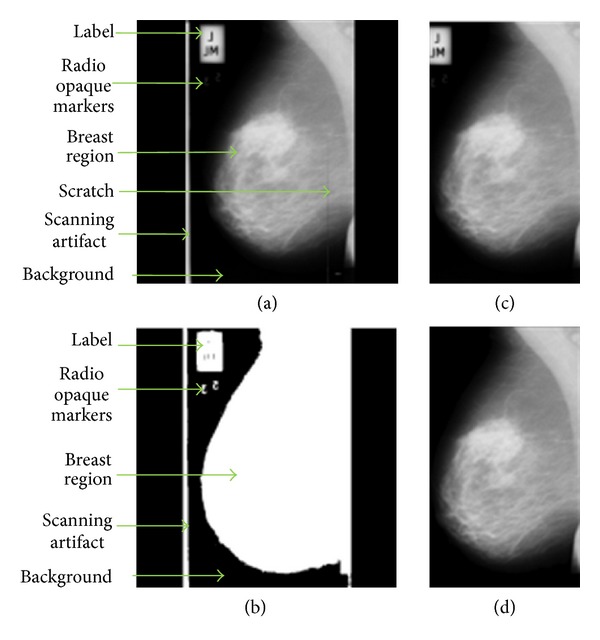
(a) Original mammogram image of mdb111; (b) binarized image of the breast region after thresholding; image after preprocessing using the median filter and automatic cropping (c) before and (d) after label and marker removal resulting in the segmented breast profile.

The segmentation technique has been tested on normal and abnormal images of mini-MIAS database. Figures 6 and 7 show the results of the preprocessing stage of this research for normal and abnormal images. The original mammogram images (Figures 6(a) and 7(a)) consist of a large background area. Thresholding technique is used to separate the breast region from the non-breast region. The binary images of breast region are shown in Figures 6(b) and 7(b). The automatic cropping will limit the mammogram images to be fed into the rectangular area of the breast region as seen in Figures 6(c) and 7(c). The morphological technique is adopted in order to remove radio opaque markers and labels. The median filter is used to enhance the image and to remove noise such as scratches in the original mammogram images, and the results of filtering are shown in Figures 6(c) and 7(c). Figures 6(d) and 7(d) show the segmented breast profile as region of interest for original image Figures 6(a) and 7(a) after removing the radio opaque marker and label. 

**Figure 8:**
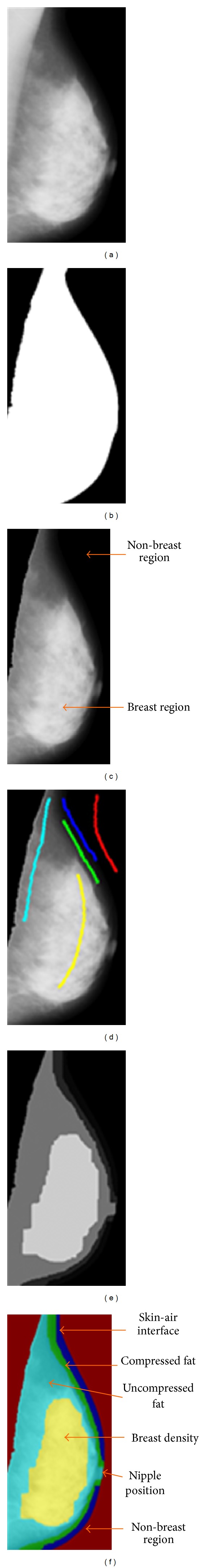
(a) The cropping breast profile image of mdb004 for right MLO mammogram, (b) binarized image of the breast region after removal of pectoral muscle, (c) mammogram image with pectoral muscle removed and segmentation of mammogram image mdb004, (d) using 5 marked labels and output segmented image with (e) grayscale label and (f) color label.

**Figure 9:**
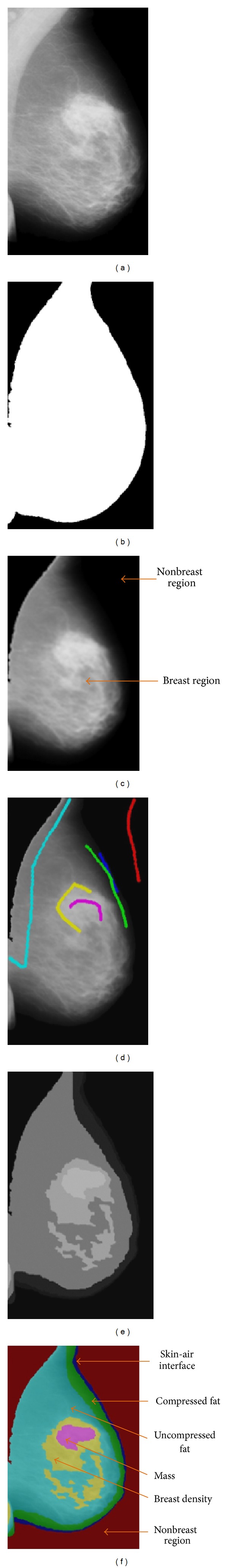
(a) The cropping breast profile image of mdb111 for left MLO mammogram is flipped horizontally, (b) binarized image of the breast region after removal of pectoral muscle, (c) gray level mammogram image with pectoral muscle removed and segmentation of mammogram image mdb111, (d) using 6 marked labels and output segmented image with (e) grayscale label and (f) color label.

Region growing is performed with initialization seed, which is allocated at the upper-left corner of the image. The right MLO mammogram image of mdb004 can be processed directly without the need to be flipped (Figure 8(a)), while the left MLO mammogram image of mdb111 has been flipped horizontally to position the pectoral muscle at the upper-left corner of the image (Figure 9(a)). Then, the pectoral muscle areas are extracted. The breast regions without pectoral muscle (Figures 7(c) and 9(c)) are used as input for segmentation using the GC technique. 

Multilabel GC technique can delineate a normal mammogram image into area of density and other breast anatomical regions. The GCs are applied with multiselection of labels. There are five labels selected for mdb004 image. The labels are marked by the user. The results of image mdb004, which is a normal case, after it is segmented using the GC technique are shown in Figures 8(d)–8(f). The first label is for finding the background, the second for finding the skin-air interface, the third for finding the uncompressed fatty region, the fourth for finding the compressed fatty region, and the fifth for finding the breast density region. For every image, there are three output images. The first image shows marked seeds by the user (Figure 8(d)) and the second shows segmentation output in the grayscale (Figure 8(e)), while the third image shows the segmentation output in color (Figure 8(f)).

The result of mdb111 which is a malignant case, after segmenting using the GC technique is shown in Figures 9(d)–9(f). Six labels are selected for the image mdb111. The first label for finding the background, the second for finding the skin-air interface, the third for finding the uncompressed fatty region, the fourth for finding the compressed fatty region, the fifth for finding the breast density region, and the sixth label is for finding the dense tumor or mass. The results showed that GC technique has the capability to detect masses which are embedded in the breast density. By focusing on the dense areas and highlighting the hyperdense regions of the glandular area, the GC technique can automatically detect the presence of mass. This research proved the importance of detecting hyperdense structures of the dense breast regions which can automatically highlight the presence of abnormalities such as a mass or masses. Quantitative evaluation of segmented breast density by the proposed method is based on the ground truths by our radiologist. 

**Figure 10:**
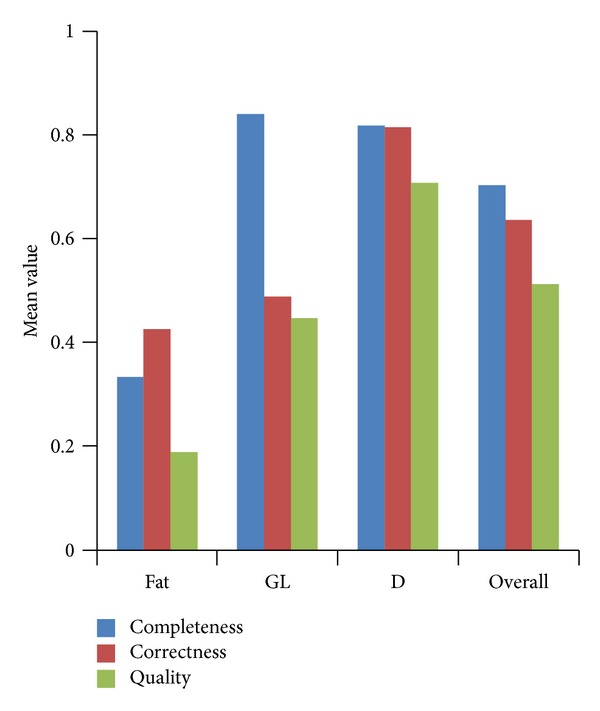
Performance evaluation results of GC using completeness, correctness, and quality techniques for different breast tissue types and overall image.

Performance evaluation has been evaluated on 40 mammograms images of different breast tissue types or BIRADS categories, with 10 images each for fatty (fat) and fatty-glandular (GL) and 20 images for dense-glandular (*D*) breast type. Overall, for the 40 normal images, the mean values for completeness (CM), correctness (CR), and quality (*ρ*) were 0.702, 0.635, and 0.513. For fatty breast tissue type, the mean values for completeness, correctness, and quality were 0.334, 0.426, and 0.189. For fatty-glandular breast tissue type, the mean values for completeness, correctness, and quality were 0.839, 0.488, and 0.447. For dense-glandular breast tissue type, the mean values for completeness, correctness, and quality were 0.818, 0.814, and 0.707. Performance evaluation results of GC technique are shown on Figure 10, where the *y*-axis represents the mean values obtained using the completeness, correctness, and quality metrics for the different breast tissue types and the overall image represented along the *x*-axis.

The performance evaluation result is promising for breast density and mass area detection. The ground truths based on the information from mini-Mias database gave the rough ground truths, while the ground truths from our radiologist gave more precision. The ground truth from mini-Mias database can provide the location of abnormality as reference. However, it cannot provide precise edge of the mass compared with ground truth used in this study. As a result, the performance evaluation conducted in this study can supply more accurate performance evaluation results. The problem regarding the ground truth and performance evaluation has been explained in detail in our review paper [27]. 

According to Nishikawa et al. [39], it is not meaningful to compare different techniques if the techniques are tested on different databases. However, there are very few techniques that have been tested using quantitative performance evaluation involving ground truth from a radiologist. Therefore, comparison has been made between our technique with other previous works using images from the same databases and the performance evaluation is conducted using the same metric, involving ground truths from a radiologist. In this study, we have compared our findings with previous work by Adel et al. [23], whereby Bayesian technique with an adaptation of Markov random field was applied and quality metric was used in their performance evaluation. The results for mdb003 (BIRAD 3), mdb041 (BIRAD 2), and mdb009 (BIRAD 1) are presented in Table 1. For the BIRAD 3 (mdb003) and BIRAD 2 (mdb041) breast categories, our method produced better results of quality metrics compared with the previous method. Although our results are found to be poor for the BIRAD 1 (mdb009), the segmented output image is quite similar, that is, if the segmented image in this study is compared with the previous segmented image by Adel et al. [23]. This means that the difference in quality metrics is caused by the difference in the ground truths. The reason is that the more detailed ground truth of glandular breast type involves multiple locations of dense area representing the heterogeneous region, while previous research paper have only considered one location spread over a large area as their ground truths. The different ground truths will affect the performance evaluation result, albeit the segmentation results are quite similar. The different ground truths greatly affect the reliability of the performance evaluation of the segmentation results. Therefore, it is absolutely necessary to find a way to obtain an objective ground truth.

As stated before, the focus of this research is not only for the segmentation of dense areas but also considers the other breast anatomical structure for visualization such as the location of nipple (if necessary and possible), the skin-air interface, uncompressed fatty tissue, compressed fatty tissue, and glandular tissue. The results indicate that the GCs technique can delineate the breast density, mass, and other breast anatomical regions in mammogram.

**Figure 11:**
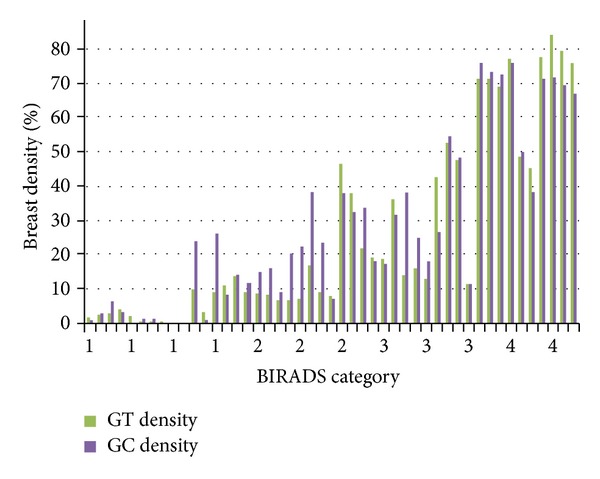
Breast density percentages of ground truths by radiologist (GT density) and breast density segmented using graph cuts (GC density).

For the calculation of breast density percentage, the number of density pixels is divided by the total number of pixels within the breast boundary. However, there is uncertainty in the definition of breast boundary. Most studies ignore the skin-air interface as breast region. Moreover, according to Karssemeijer [21], it is important to include the skin-air interface and also to preserve nipple position (if possible) for feature selection. Therefore, this research has considered these aspects in order to provide more precise segmentation. Pearson correlation coefficient (*r*) was used to compare the dense breast areas segmented using GC method with the breast density area derived by the ground truths in this research. The results is robust (*r* = 0.93) and comparable with previous work by Zhou et al. (*r* = 0.91 and *r* = 0.94) [15].  Figure 11 shows the breast density percentages of ground truths by our radiologist (GT density) and breast density segmented using GC (GC density).

The results show that the percentages of the segmented area using GC highly correlate with the segmented area by the radiologist. However, a lower percentage is produced as compared to the category that is derived by BIRADS. This is caused by the different definitions of breast boundary. Previous research defined breast boundary without considering the skin-air interface [20], because of the difficulty in visualizing that area, as a result of low contrast. On the other hand, this study which has emphasized that the anatomical breast region has not discarded this skin-air interface region as it helps in the visualizating and interpreting purpose. However, this has decreased the density percentage, while the breast region area has become larger. The situation causes bias in categorizing breast density. In order to overcome this situation, a model based on statistical analysis using regression analysis is performed. A model to determine breast density category based on breast density percentage is derived as follows:

(4) $$\begin{array}{c} y=1.327+0.040\left(x\right), \end{array}$$

where *y* is the estimated BIRADS category while *x* is the density percentage.

## 5. Discussion

The preprocessing stage which involved automatic breast profile cropping can identify regions of interest (ROI), in the breast. It also eliminated unnecessary areas, such as the large background area, radio opaque markers, labels, and artifacts. The segmentation technique is able to segment the identified dense breast areas, and its capability in detecting mass embedded in the dense areas is highlighted. The method has also helped in the visualization of other breast anatomical regions. A strong correlation of *r* = 0.93 was observed between the segmented breast density and the radiological ground truth. The promising results showed the potential capability of the technique to be used in clinical practice for quantifying breast density.

Two situations arise using the proposed approach. Firstly, the completeness, correctness, and quality of the proposed method are good or better for dense breast type (BIRADS 4). This is because the homogenous region needed one sketching of ground truth. However, as a result of using the detailed and precise ground truth, the completeness, correctness, and quality of the proposed method are lower for the fatty and glandular breast type. This is because the more detailed ground truth of glandular breast type involves multiple locations of dense areas to represent the heterogeneous region and is not confined to only one location as in the previous ground truths [35]. Only one sketch of ground truths in this research is used for the homogenous region. It is very challenging to compare manually drawn ground truths sketching with the computerized result. This is because the manually drawn ground truth sketches by the radiologist are based on expert's vision of the related breast anatomical structure, while the computerized results are based on intensities. While the computer may be powerful to discriminate pixel value based on the differing intensities, the radiologists empower their naked eyes in the differentiation of intensity. However, based on his experience and instinct, a radiologist is able to interpret results based on detailed anatomical structures compared with a computerized method which is confined to the levels of intensities. In any case, this research has stated the necessity for the definition of ground truths of breast densities, as a guidance to be used in a repeatable manner. 

Secondly, in order to classify a breast according to BIRAD categories, the density percentage is calculated. There is high correlation (*r* = 0.93) between the breast density area segmented using GC method with the breast density area derived by the ground truth in this research. However, the value of the density percentage is smaller than its BIRAD categories. Again, this situation is difficult to solve, unless a clear definition of a ground truth and breast region is available. Therefore, a model for breast category estimation is derived using statistical analysis. As this study had focused on breast density percentage as a feature for classification using regression analysis, the classification of other features is beyond the scope of our study. Briefly, the calculation percentage of glandular tissue has been conducted, and the estimation of breast density category has been derived. 

## 6. Future Works

**Figure 12:**
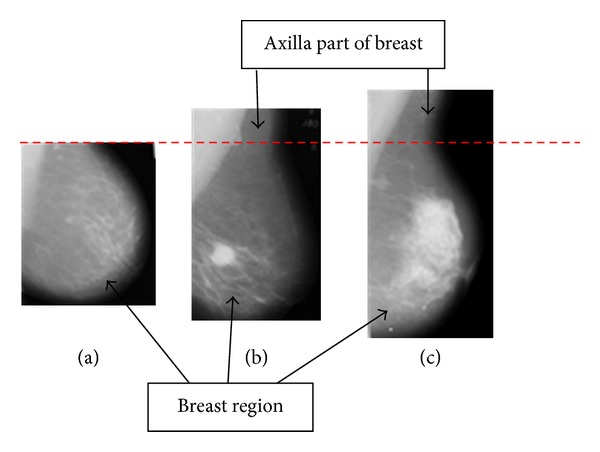
Different sizes of the axilla portion on mammogram images.

Future research should try to identify the same ground truth as a term of reference in order to compare the computer assisted system that will be developed. A standard definition or explanation of ground truth is deemed necessary, so that an objective ground truth can be sketched correctly according to the criteria derived. A clear definition of what constitutes a breast region should also be stated in future works, that is, such as whether the axilla portion as well as pectoral muscle section should also be considered. We have found that some images do not include the axilla portion (Figure 12(a)), while some other images have taken a small section of that part (Figure 12(b)), and still others have considered larger portions of the axilla (Figure 12(c)). This study suggests the removal of the axilla for future research. This is because each of the MLO mammogram images has different sizes of the axilla portion of the breast region, which will affect the calculation of the overall breast region area. Removal of the axilla section assures that the same definition of breast region could be obtained and a more standard measurement of the breast region area could be used in future research. 

This study suggests the classification of BIRAD density in future work, not only based on the percentage area of breast density but also in combination with morphological or textural features (GLCM) of breast region. The classifier (SVM, PCA, ID3, MLP, NN or other classifiers) should be used to classify the breast density into BIRAD categories. 

## 7. Conclusion

Previous research papers have focused either on breast density or mass detection. This research combined both abilities (breast density or mass detection) in a system. The system not only could segment breast density and other breast anatomical regions, it also could highlight mass or masses that may be embedded in the glandular regions. These are beneficial computational tools to be used for clinical decision support systems in the diagnosis of breast cancer. The GC algorithm which has been implemented for breast density, mass, and anatomical segmentation was found to be promising. The precision of the segmentation technique has great effects on image analysis. This study has also emphasized the usage of detailed and precise ground truths especially in the performance evaluation. A standard definition or explanation of ground truth is necessary to ascertain that correct sketches are obtained based upon agreed and derived criteria. This study has also tried to highlight the fact that comparison of performance evaluation for segmentation results by using different ground truths is incomparable until and unless a clear definition of ground truths is stated for research in the future. A clear definition of breast area is also important for the calculating of breast density. In any case, the ground truth can be accepted as guidance for performance evaluation. However, it is not an absolute evaluation. There is still restriction and limitation in the performance evaluation of segmentation results, and this needs further improvements. 

## Figures and Tables

**Table 1:** Comparison results of the proposed method with previous work by Adel et al. [23].

Segmentation technique			
Adel et al. [23]		Proposed method	
Image	Quality metric	Image	Quality metric
mdb003	0.58	mdb003	0.831
mdb041	0.77	mdb041	0.865
mdb009	0.185	mdb009	0.130
